# Supplementary material for: Microbial profile in bile from pancreatic and extra-pancreatic biliary tract cancer
Source: PLoS One. 2024 Feb 21;19(2):e0294049. doi: 10.1371/journal.pone.0294049 (PMC10880987; doi:10.1371/journal.pone.0294049)
Supplement: S1 Table — Particularly, we reported for each subgroup the patients with fungus presence, and in the last row the patients with fungus only. (DOCX) [file pone.0294049.s001.docx]

**S1 Table.** Gram-negative, positive and both isolated composition in PC group. Particularly, we reported for each subgroup the patients with fungus presence, and in the last row the patients with fungus only.

| **Nr. Patients** | **Age** | **Gender** | **Group** | **Bacteria** |
| --- | --- | --- | --- | --- |
| **Gram-** | | | | |
| n=3 | 75.3±6.1 | 1M, 2F | PC | *Achromobacter spp* |
| n=6 | 76.3±14.3 | 4M, 2F | PC | *Acinetobacter spp* |
| n=2 | 70.0±15.6 | 2M, 0F | PC | *Acinetobacter spp,*  *Pseudomonas spp,*  *Stenotrophomonas spp* |
| n=1 | 89 | M | PC | *Brevundimonas spp* |
| n=5  n=1/5 | 79.4±5.0  75 | 3M, 2F  F | PC  PC | *Citrobacter spp*  *Candida spp* |
| n=2 | 75.5±4.9 | 1M, 1F | PC | *Delftia acidovorans* |
| n=1 | 74 | F | PC | *Enterobacter spp* |
| n=14 | 74.2±11.8 | 8M, 6F | PC | *Escherichia coli* |
| n=1 | 79 | F | PC | *Escherichia coli*  *Achromobacter spp*  *Candida spp* |
| n=11 | 74.5±10.8 | 5M, 6F | PC | *Klebsiella spp* |
| n=1 | 80 | M | PC | *Klebsiella spp*  *Escherichia coli*  *Candida spp* |
| n=22 | 77.1±9.3 | 13M, 9F | PC | *Pseudomonas spp* |
| n=1 | 53 | M | PC | *Pseudomonas spp,*  *Stenotrophomonas spp,*  *Klebsiella spp* |
| n=1 | 54 | F | PC | *Pseudomonas spp,*  *Elizabethkingia meningoseptica* |
| n=1 | 57 | M | PC | *Pseudomonas spp,*  *Escherichia coli* |
| n=5 | 73.8±11.7 | 2M, 3F | PC | *Stenotrophomonas spp* |
| ***Gram+*** | | | | |
| n=1 | 87 | F | PC | *Staphilococcus spp* |
| n=2 | 83.5±0.71 | 2M, 0F | PC | *Enterococcus spp* |
| ***Gram+* and *Gram-*** | | | | |
| n=1 | 81 | M | PC | *Citrobacter spp*  *Enterococcus spp* |
| n=1 | 94 | M | PC | *Pseudomonas spp*  *Enterococcus spp* |
| n=1 | 81 | M | PC | *Enterobacter spp*  *Enterococcus spp*  *Candida spp* |
| n=3  n=3/3 | 82.0±7.0  82.0±7.0 | 2M, 1F  2M, 1F | PC  PC | *Escherichia coli*  *Enterococcus spp*  *Candida spp* |
| n=1 | 70 | F | PC | *Sternotrophomonas spp, Klebsiella spp*  *Enterococcus spp* |
| n=1 | 78 | F | PC | *Pseudomonas spp Stenotrophomonas spp*  *Enterococcus spp* |
| n=1 | 79 | M | PC | *Escherichia coli*  *Serratia spp*  *Streptococcus spp Enterococcus spp* |
| n=1 | 58 | F | PC | *Achromobacter spp*  *Stenotrophomonas spp*  *Streptococcus spp* |
| ***Fungus*** | | | | |
| n=4 | 81.8±10.2 | 2M, 2F | PC | *Candida spp* |
