# Supplementary material for: Microbial profile in bile from pancreatic and extra-pancreatic biliary tract cancer
Source: PLoS One. 2024 Feb 21;19(2):e0294049. doi: 10.1371/journal.pone.0294049 (PMC10880987; doi:10.1371/journal.pone.0294049)
Supplement: S2 Table — Particularly, we reported for each subgroup the patients with fungus presence, and in the last row the patients with fungus only. (DOCX) [file pone.0294049.s002.docx]

| **S2 Table.** Gram-negative, positive and both isolated composition in EPC group. Particularly, we reported for each subgroup the patients with fungus presence, and in the last row the patients with fungus only. | | | | |
| --- | --- | --- | --- | --- |
| **Nr. Patients** | **Age** | **Gender** | **Group** | **Bacteria** |
| ***Gram-*** | | | | |
| n=3 | 75.3±12.5 | 2M, 1F | EPC | *Achromobacter spp* |
| n=2 | 76.0±15.6 | 1M, 1F | EPC | *Brevundimonas spp* |
| n=1 | 72 | F | EPC | *Citrobacter spp*  *Pseudomonas spp* |
| n=1 | 49 | F | EPC | *Delftia spp* |
| n=4 | 75.8±2.2 | 4M, 0F | EPC | *Enterobacter spp* |
| n=4 | 73.8±7.0 | 2M, 2F | EPC | *Alcaligenes fecalis* |
| n=1 | 93 | M | EPC | *Elizabethkingia meningoseptica* |
| n=11 | 69.3±7.8 | 4M, 7F | EPC | *Escherichia coli* |
| n=1 | 70 | F | EPC | *Escherichia coli*  *Citrobacter spp*  *Klebsiella spp*  *Morganella morganii*  *Candida spp* |
| n=2 | 76.5±13.4 | 1M, 1F | EPC | *Klebsiella spp* |
| n=9 | 71.1±6.7 | 5M, 4F | EPC | *Pseudomonas spp* |
| n=2 | 81.0±1.4 | 1M, 1F | EPC | *Pseudomonas spp*  *Klebsiella spp* |
| n=1 | 73 | F | EPC | *Stenotrophomonas spp* |
| n=1 | 84 | M | EPC | *Stenotrophomonas spp,*  *Pantoea agglomerans* |
| ***Gram+*** | | | | |
| n=2 | 77.5±0.7 | 1M, 1F | EPC | *Enterococcus spp* |
| ***Gram+* and *Gram-*** | | | | |
| n=1 | 68 | F | EPC | *Stenotrophomonas spp*  *Enterococcus spp* |
| n=2 | 80.0±9.9 | 2M, 0F | EPC | *Pseudomonas spp*  *Enterococcus spp* |
| n=1 | 79 | F | EPC | *Enterobacter spp*  *Enterococcus spp* |
| n=1 | 84 | F | EPC | *Pseudomonas spp*  *Serratia spp*  *Enterococcus spp* |
| n=1 | 62 | M | EPC | *Citrobacter spp Pseudomonas spp Enterococcus spp* |
| ***Fungus*** | | | | |
| ⎯ | ⎯ | ⎯ | ⎯ | ⎯ |
